# Brazilian Samba Protocol for Individuals With Parkinson’s Disease: A Clinical Non-Randomized Study

**DOI:** 10.2196/resprot.6489

**Published:** 2017-07-04

**Authors:** Ana Cristina Tillmann, Alexandro Andrade, Alessandra Swarowsky, Adriana Coutinho De Azevedo Guimarães

**Affiliations:** ^1^ Health Sciences Centre (CEFID) Research Laboratory in Leisure and Physical Activity (LAPLAF) University of Santa Catarina State Florianópolis Brazil; ^2^ Health Sciences Centre (CEFID) University of Santa Catarina State Florianopolis Brazil

**Keywords:** Parkinson's disease, protocol, balance, quality of life, therapy

## Abstract

**Background:**

In the 10 most populated countries in the world, Parkinson's disease (PD) affects more than 5 million individuals. Despite optimal treatment options already developed for the disease, concomitant involvement of other areas of health care plays an important role in complementing the treatment. From this perspective, dancing can be viewed as a non-drug alternative that can reduce falls by improving some motor skills, such as mobility, balance, gait, and posture, and can also improve the overall quality of life. Brazilian samba promotes improvement in motor and non-motor symptoms in individuals with PD, providing a new treatment option for this population.

**Objective:**

The main objective of this quasi-experimental study is to provide a 12-week samba protocol (2x/week) for individuals with PD and to compare its effects with the group without intervention. The hypothesis is that the Brazilian samba protocol will promote improvement in primary (motor) and secondary (non-motor) outcomes in individuals with PD.

**Methods:**

The sample will be selected at random from individuals diagnosed with PD in the city of Florianopolis (SC, Brazil). Sample size calculation was performed with the G*Power 3.1.9.2 software, with 0.447 effect size, at 5% significance level, power of 0.9, and test and sample loss of 20%. This yielded 60 individuals divided between the intervention and control groups. The questionnaires will be filled out before and after the dance intervention. The data collection for the control group will be held simultaneously to the intervention group. The classes will last for 1 hour, twice a week in the evening for 12 weeks, and all classes will be divided into warm-up, main part, and relaxation. Two-way analysis of variance with repeated measures and Sidak post-hoc comparison test will be used for a comparative analysis of the final results of the control group with the experimental group and of the within-group changes between pre- and postintervention period.

**Results:**

We expect to complete follow-up in September 2017.

**Conclusions:**

The major inspiration for this study was to encourage the creation of new rehabilitation programs that do not emphasize doctor involvement. This is a unique protocol for PD and we believe it can be an important tool to alleviate the motor and non-motor symptoms of individuals with PD. Dance is a simple activity depending on little equipment and few financial resources, facilitating its implementation and improving the cost-benefit relationship. In addition, activities that have a cultural aspect for the population in question, and which are pleasant, enable the participants to commit long term. This can enhance patient’s compliance with the therapy, which is often a problem for many rehabilitation programs.

## Introduction

In the 10 most populated countries in the world, Parkinson’s disease (PD) affects more than 5 million individuals and the projection is that by 2030 more than 9.3 million people will have been diagnosed [1]. Given its epidemiological importance, the disease can be considered a public health problem, manifestations of which encompass social, family, economic, and even legal problems [2], and involve high costs to the health system. Individuals with chronic diseases, such as PD, deal with physical discomforts on a daily basis arising from deficits and motor symptoms [3-6] and as autonomic, sensory, and behavioral disorders from non-motor symptoms. These can result in a severe reduction of independence [7] and quality of life in this population [8].

From this perspective, dancing can be viewed as a non-drug alternative for both symptomatologies: (1) improving some motor skills (eg, mobility, balance, gait, posture, fall reduction), and (2) improving some non-motor symptoms (eg, fatigue and depression). The benefit is an overall improvement in quality of life [9-11]. For this population, dance is perhaps the most “complete” of all interventions, mainly because the stimulation of different cerebral areas is so important for patients with PD.

Recently, Sharp et al [12] conducted a systematic review, which sought to analyze the effectiveness of dance as an intervention for PD. They found improvement in motor scores on Unified Parkinson’s Disease Rating Scale (UPDRS), balance, and gait speed in subjects who underwent dance therapy compared to the group that did not receive any intervention. Similarly, there was an improvement in quality of life when dance intervention was compared to exercise interventions [12]. In another systematic review, dance demonstrated maintenance of muscle strength and improvements in support and balance, aerobic power, full range of body movements, resulting in positive lifestyle changes in individuals with PD [13].

Among the surveys already performed with dance and patients with PD, tango is the most commonly applied ballroom dance form in studies involving PD [12]. According to Earhart [14], the practice of this particular rhythm could facilitate the activation of brain areas that normally are less active in PD. Tango movements, following a well-defined and precise rhythm, were associated with increased activation of neural areas not normally active in patients with PD [15] and with stimulation of cortical activation by increasing motor skills [16]. These results indicate that the characteristics of this rhythm may increase the demand for conscious awareness of movement in patients with PD, thus facilitating mobility [14].

Widespread in Latin America, especially in Brazil, the samba is a dance that evolved similar to tango. It was influenced by African rhythms and became popular in Europe at the same time, which explains the similarity in the execution of steps in the two rhythms [17]. Samba has a two-four time rhythm and has similar passages and diversity of execution as tango. Movement can be performed with higher or lower speed without necessarily being out of the musical time [18]. Samba marking characteristics and basic step, with stops in the movements and shifts forward and back, are similar to tango markings [19].

In addition, samba has many lively rhythms and makes use of percussion instruments, stimulating its dancers, facilitating awareness, and marking the rhythm. There are numerous styles of samba, like the slower bossa nova, or the faster gafieiras sambas [20]. This rhythm has already been used in the area of rehabilitation, with patients recovering from heart disease. It was shown that participants adapted well to the samba protocol, and it was feasible as possible exercise during cardiac rehabilitation [18].

Although the tango world is widespread, use of other dance styles could lead to more accessible and attractive interventions, when adapted to culture of the population. Furthermore, it may facilitate diversity of music [21] and application protocols. However, there is still a lack of evidence of the effectiveness of rhythms other than tango in PD. Thus, the main objective of this non-randomized study is to provide a 12-week samba protocol (2x/week) for individuals with PD and to compare its effects with the group without intervention. We hypothesize that the Brazilian samba protocol will promote improvement in primary (motor) and secondary (non-motor) outcomes in individuals with PD, providing a new treatment option for this population.

## Methods

### Study Type

**Figure 1:**
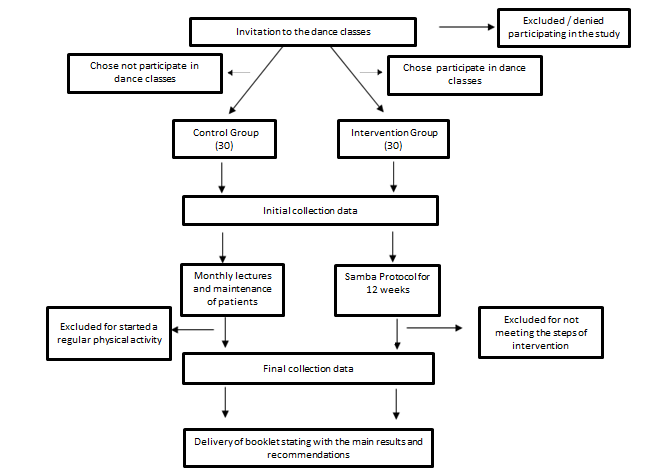
Flowchart showing the process of participant selection and steps in the study protocol.

This clinical non-randomized study comprises 12 weeks of intervention and monitoring of a control group (Figure 1).

### Participants

The sample will be selected at random from individuals diagnosed with PD in the city of Florianopolis (SC, Brazil). They will be recruited from a telephone list of individuals who take part in the Parkinson Association Santa Catarina. Half of those (n=30, intervention group) will be take part in activities in the Rhythm and Movement Extension Program at the Health Sciences and Sports Centre at the State University of Santa Catarina in the Santa Catarina Rehabilitation Center (CCR).

Inclusion criteria consist of individuals of both sexes; aged ≥50 years; with a clinical diagnosis of PD, according to the criteria of the London Brain Bank [22]; on stable doses of medication; who attend one or two times weekly, a physiotherapy section and who have not participated in any dance classes for at least 3 months. Exclusion criteria consist of people who are taking part in combined practice of any physical activity program and/or exercise; fail to complete all stages of the study (intervention); are decompensated clinically; do not meet the cut-off points on the Mini-Mental State Examination, in accordance with the criteria established by Bertolucci et al [23] for the Brazilian population (13=illiterate, 18=medium level of education, 26=high school); and with physical disability affecting daily or social life activities, due to a condition other than PD.

In order to avoid confusion bias before the intervention, participants will be excluded from either of the groups, if they have participated in systematic physical activity, for at least 3 months prior to the start of the study.

### Outcome Measures

According to interventions already undertaken with distinctive dance forms [9,21,24-27], it is believed that the samba classes can generate positive results in both motor symptoms and non-motor symptoms. The expectation is that the activity will have a greater effect on the primary outcomes such as motor exploration and balance, and a minor effect on non-motor symptoms such as depression and fatigue. We also decided to include an assessment for sleep disorders, as this outcome is still not observed in dance studies. To maintain uniformity in the results of future studies, we chose the more popular assessment tools for each variables, as well as the number of outcomes. Table 1 lists the variables to be evaluated, as well as the instruments used to measure them.

### Ethical Approval

The project was approved by the Research Ethics Committee in Human Beings, University of Santa Catarina State under the protocol 1.268.353.

**Table 1:** Variables and instruments used for assessment of the outcomes in individuals with PD.

Variables		Definition	Instruments
**Symptomatology**				
	General condition of the patient with PD	Light disability, Moderate disability, High disability	HY^a^-Degree of Disability Scale
	Evaluation of PD	Progression of the disease	UPDRS^b^	
**Motor/Non-motor Symptoms**				
	Balance	Restricted to the wheelchair; Needs during walking; Independent walking	BBS^c^
	Sleep quality	‒	PDSS^d^
	Depressive symptoms	Minimal, mild, moderate, severe	BDI^e^
	Fatigue	‒	FSS^f^
	Quality of life	‒	PDQ-39^g^

^a^HY: Hoehn and Yahr.

^b^Unified Parkinson's Disease Rating Scale.

^c^BBS: Berg Balance Scale.

^d^PDSS: Parkinson’s Disease Sleep Scale.

^e^BDI: Beck Depression Inventory.

^f^FSS: Fatigue Severity Scale.

^g^PDQ-39: Parkinson’s Disease Questionnaire.

### Data Collection

#### Experimental Group

The classes will be held at the CCR while participants are taking their medication. They will be invited to participate in the study voluntarily and to take part in the dance classes. The potential participants will be provided with an explanation of the study procedures (assessment prior to data collection, dance classes, and assessment after data collection) and the importance of attending the classes for health benefits. After the participants sign the informed consent forms, we will start collecting data. The questionnaires will be filled out before the dance intervention in a quiet place to facilitate subjects’ understanding of the questions, during a 2-week period preceding the application of the protocol. After the initial assessment, the dance classes will start twice a week and last 1 hour each time, according to the study protocol. At the end of the 12-week course, a second assessment will be held, in the same manner as the first one.

#### Control Group

The data collection for the control group will be held simultaneously to the intervention group of the period. The researcher will schedule a visit to the patient’s home or other preferred place, where they will clarify the goals of the study, emphasize the importance of keeping to normal daily activities, and request that the patient avoids starting a physical activity during these 12 weeks. The subjects in the control group will also be advised that not performing physical activity for 12 weeks is against the guidance for prevention of disease and maintenance of health, and therefore, it is only a guideline.

The participants in the control group will be invited to monthly lectures that address the maintenance of health, prevention of falls, and psychological care. They will be asked, in person, about their general health and performance of daily activities. During the interview after the 12 weeks of data collection, any subjects who state that they have started a regular physical activity of any type will be excluded from the control group. At the end of the study, all participants (control and intervention) will receive a booklet stating, in a clear and objective way, the main results for both the group and individually (including the activities started during the period and the option to be excluded from the databank). Since the dance classes will continue after the end of the study, all participants will be invited to take part in them.

### Intervention: Brazilian Samba Protocol

The dance protocol will consist of dance classes, which will be conducted in a spacious room with appropriate facilities for a dance class for individuals with PD (floor without deformities, chairs for resting, and stereo with moderate volume to allow everyone to clearly hear the music). The classes will last for 1 hour, twice a week in the evening, for 12 weeks. The rhythm chosen for the classes is the Brazilian samba (two-four rhythm), as it was identified that its high resemblance to tango in the strong marking and the basic forward-backward steps can help development of balance and muscle tone of individuals. Furthermore, as a known national rhythm, appreciated by most of the population, it should facilitate obtaining participants’ compliance with the protocol. All classes will be divided into warm-up, main part, and relaxation. The activities will start with typical, 10-minute warm-up for ballroom dances suggested by Hackney and Earhart [9], including light walking to the rhythm of pre-selected music (samba), focusing on the breath, followed by light exercise of upper limb movement (rotation, abduction, and adduction), while always working on the musicality and rhythm, as well as postural alignment. During the main part of the lesson, the participants will follow the teacher/researcher instructions. The teacher will first demonstrate the step to be taught in that class, and the participants will initially execute it without a partner. Next, they will be paired up and perform the requested activity. The main part of the lesson will last 35 minutes, and partners will change every 7 minutes. This is aimed at facilitating practice with the largest possible number of pairings to develop motor skills and balance and to improve the adaptation to change process. During the lessons, activities will be divided into (1) following the commands of the teacher, or (2) dancing freely. The steps to be taught are described in Table 2. The sequences of classes and selected songs, including the steps, are shown in Multimedia Appendix 1.

**Table 2:** Description of the steps to be taught.

Steps	Feature execution
Presentation of Rhythm	During the class, traversing movements with songs of different speeds will be executed, to familiarize students with the rhythm.
Basics Steps	One step forward, two steps in place, and a step back.
Ginga^a^	With one foot forward and one foot back, the students shift their weight between the front and back legs without moving.
Working with Rhythm	Adapting previously learned movements and steps to songs of different rhythms and speeds.
“X”^a^	The pair forms an X, to perform the basic step forward. The gentleman goes on the side of the lady.
Cruzado^a^	Starting in the position of X, the pair faces each other and returns to the X position.
Bate e Volta^a^	Starting in the starting position, the lady and gentleman move away from each other and return to the starting position.
Steps Sequence	Students repeat the learned steps in predefined sequence, first pausing after each step, until they will are able to perform them with music.
Samba no pé^a^	Steps taken individually focusing on shifts at different rhythms.
Giro da Dama^a^	Starting with the backwards basic step, the gentleman leads the lady to make the turn, the lady holds the rotation moving forward, and the gentleman accompanies her movement walking forward at her side.
Review of Steps	At random, a review with all learned steps will be performed to enhance learning and improve step implementation.
Prom	During the free dancing session, music of different speeds and rhythms will be included. Students will be dancing together, and the pairs will be encouraged to swap with every song to practice the already-learned steps.

^a^The steps names were maintained in their original language.

The songs were divided, according to the beats per minute (bpm), into three movements: slow (40-72 bpm), medium (72-120 bpm), and fast (120-208 bpm) [18]. The beats analysis was performed with BPM Detector Pro.

At the end of every class, calm exercises will be performed for 5 minutes to promote relaxation after activity, involving massage, stretching, or a slow walk with quiet music in the background.

### Statistical Analysis and Sample Size

Sample size was based on primary outcomes and calculation performed with the G*Power 3.1.9.2 software [28], assuming a moderate effect of Brazilian samba on motor signals (UPDRS III), according to Cohen [29] with 0.447 effect size. We chose a more cautious effect size because there is still no evidence of Brazilian samba in DP rehabilitation. However, due to the effects already found in similar interventions (ref=-0.62/-0.55) [30] as described in the meta-analysis by Lutzke et al, a significant effect is expected regarding to motor symptoms. We used alpha=.05 and power of 0.9, applied in two groups (experimental group and control group) analyzing the results through a two-way analysis of variance (ANOVA) with repeated measures and Sidak post-hoc comparison test for a comparative analysis into and between groups. Significance level will be set at 5%. Anticipating 25 individuals in each group, but foreseeing a sample loss of around 20% [31,32], we have chosen to include 30 individuals per group, ending with a total of 60 subjects.

After the intervention, the data will be transferred to SPSS v. 20.0. First, descriptive statistics will be performed (mean, standard deviation, and percentage). Two-way ANOVA with repeated measures and Sidak post-hoc comparison test will be used for a comparative analysis of the results of the control group with the experimental group and of the within-group changes between pre- and postintervention period. Significance level will be set at 5%.

## Discussion

### Principal Findings

The major inspiration for this study was to encourage the creation of new rehabilitation programs that do not emphasize doctor involvement. A further goal was to provide an environment where patients, caregivers, and teachers can interact in a friendly and relaxed manner, while stimulating and encouraging social relations, important for maintaining of the well-being of patients [21].

Dance, already used as a rehabilitation tool with other diseases [33,34], has properties that address important aspects of PD [12,13]: music stimulates affective memory and the rhythm engages different neural regions [35]. Dance replaces treatments or exercises that sometimes discourage patients. In addition, because it embraces natural movements to the human being [36], dance stimulates control and motor actions, which are of great value to individuals with PD [10-13].

The positive results already found in similar studies lead us to believe in the potential of this intervention for these individuals. The protocol’s main characteristics are based on four pillars: gradual progression, stimulation of adaptability, constant revision, and provision of a lively environment during the activities. The first two pillars mainly involve motor symptoms such as gait control and balance, while the last two pillars stimulate non-motor symptoms such as cognitive stimulation and memory, providing an enjoyable activity leading to a reduction in fatigue and depressive symptoms, in addition to stimulating social interaction.

The gradual progression of difficulty of the exercises will facilitate learning and give students the motivation to learn new steps. Introduction of new steps every 2 weeks (except when the turns are taught) is extremely important in PD because the motor skills essential to daily activities often require adaptability to changing environments and the ability to deal with unpredictable fluctuations in the disease. Therefore, expanding the motor skills repertoire must be constant [9].

Further, stimulating memory function is no less important than stimulating adaptability. The process of constantly reviewing the steps already learned will promote the exercise of cognitive abilities, instigating memory formation, and activation of neural areas that normally have a reduced activity in PD [15,37]. The basic samba steps, characterized by steps forward and back and performed rhythmically, can stimulate cortical activation, as has been observed in tango, increasing the attention and concentration, especially in regards to mobility [14].

### Conclusions

This is a unique protocol for individuals with PD, which can be an important tool to alleviate motor and non-motor symptoms. Currently, validated study protocols for this population are geared mostly to exercises for specific motor tasks [38,39], ways to run long-term monitoring and management of the patients [40,41], or even different types of dance [42]. Dance is a simple activity depending on little equipment and few financial resources, facilitating its implementation and improving the cost-benefit relationship. In addition, activities that have a cultural aspect for the population in question, and which are pleasant, enable the participants to commit long term. This can enhance patient’s compliance with therapy, which is often a problem for many rehabilitation programs [14].

While these benefits are important on the international scene, they present great value in countries similar to Brazil, which faces challenges in funding disease treatment and has limited investments for research in rehabilitation.

## Appendix

Multimedia Appendix 1 Brazilian samba protocol for 12 weeks to PD patients.

## Abbreviations

BBS: Berg Balance Scale
BDI: Beck Depression Inventory
Bpm: beats per minute
CCR: Health Sciences and Sports Centers at State University of Santa Catarina in Santa Catarina Rehabilitation Center
FSS: Fatigue Severity Scale
HY: Hoehn and Yahr
PD: Parkinson’s disease
PDQ-39: Parkinson’s Disease Questionnaire
PDSS: Parkinson’s Disease Sleep Scale
UPDRS: Unified Parkinson’s Disease Rating Scale

## References

[1] Dorsey ER, Constantinescu R, Thompson JP, Biglan KM, Holloway RG, Kieburtz K, Marshall FJ, Ravina BM, Schifitto G, Siderowf A, Tanner CM (2007). Projected number of people with Parkinson disease in the most populous nations, 2005 through 2030. Neurology.

[2] Corvol JC (2014). Addictions comportementales dans la maladie deParkinson. Bull Acad Natl Med.

[3] Eggers C, Kahraman D, Fink GR, Schmidt M, Timmermann L (2011). Akinetic-rigid and tremor-dominant Parkinson's disease patients show different patterns of FP-CIT single photon emission computed tomography. Mov Disord.

[4] Lewis MM, Du G, Sen S, Kawaguchi A, Truong Y, Lee S, Mailman RB, Huang X (2011). Differential involvement of striato- and cerebello-thalamo-cortical pathways in tremor- and akinetic/rigid-predominant Parkinson's disease. Neuroscience.

[5] Helmich RC, Hallett M, Deuschl G, Toni I, Bloem BR (2012). Cerebral causes and consequences of parkinsonian resting tremor: a tale of two circuits?. Brain.

[6] Lee HK, Altmann LJP, McFarland N, Hass CJ (2016). The relationship between balance confidence and control in individuals with Parkinson's disease. Parkinsonism Relat Disord.

[7] Prakash KM, Nadkarni NV, Lye W, Yong M, Tan E (2016). The impact of non-motor symptoms on the quality of life of Parkinson's disease patients: a longitudinal study. Eur J Neurol.

[8] A'Campo LEI, Spliethoff-Kamminga NGA, Roos RAC (2011). An evaluation of the patient education programme for Parkinson's disease in clinical practice. Int J Clin Pract.

[9] Hackney ME, Earhart GM (2010). Recommendations for Implementing Tango Classes for Persons with Parkinson Disease. Am J Dance Ther.

[10] Rios RS, Anang J, Fereshtehnejad S, Pelletier A, Postuma R (2015). Tango for treatment of motor and non-motor manifestations in Parkinson's disease: a randomized control study. Complement Ther Med.

[11] Westheimer O, McRae C, Henchcliffe C, Fesharaki A, Glazman S, Ene H, Bodis-Wollner I (2015). Dance for PD: a preliminary investigation of effects on motor function and quality of life among persons with Parkinson's disease (PD). J Neural Transm (Vienna).

[12] Sharp K, Hewitt J (2014). Dance as an intervention for people with Parkinson's disease: a systematic review and meta-analysis. Neurosci Biobehav Rev.

[13] Shanahan J, Morris ME, Bhriain ON, Saunders J, Clifford AM (2015). Dance for people with Parkinson disease: what is the evidence telling us?. Arch Phys Med Rehabil.

[14] Earhart GM (2009). Dance as therapy for individuals with Parkinson disease. Eur J Phys Rehabil Med.

[15] Brown S, Martinez MJ, Parsons LM (2006). The neural basis of human dance. Cereb Cortex.

[16] Sacco K, Cauda F, Cerliani L, Mate D, Duca S, Geminiani GC (2006). Motor imagery of walking following training in locomotor attention. The effect of “the tango lesson”. Neuroimage.

[17] Ried B, Ried B (2004). Fundamentos da dança de salão.

[18] Braga H, Gonzáles A, Sties S, Carvalho G, Netto A, Campos O, Lima D, Carvalho T (2015). The brasilian samba protocol for cardiac rehabilitation. Rev Bras Med Esporte.

[19] Perna M (2005). Samba de Gafieira - a História da Dança de Salão Brasileira.

[20] Jost M (2015). A construção/invenção do samba: Mediações e interações estratégicas. Rev Inst Estud Bras.

[21] McNeely ME, Mai MM, Duncan RP, Earhart GM (2015). Differential Effects of Tango Versus Dance for PD in Parkinson Disease. Front Aging Neurosci.

[22] Hughes AJ, Daniel SE, Kilford L, Lees AJ (1992). Accuracy of clinical diagnosis of idiopathic Parkinson's disease: a clinico-pathological study of 100 cases. J Neurol Neurosurg Psychiatry.

[23] Bertolucci P, Brucki S, Campacci S, Juliano Y (1994). The Mini-Mental State Examination in an outpatient population: influence of literacy. Arq Neuro-Psiquiatr.

[24] Hackney ME, Kantorovich S, Levin R, Earhart GM (2007). Effects of tango on functional mobility in Parkinson's disease: a preliminary study. J Neurol Phys Ther.

[25] Hackney ME, Earhart GM (2009). Short duration, intensive tango dancing for Parkinson disease: an uncontrolled pilot study. Complement Ther Med.

[26] McGill A, Houston S, Lee RY (2014). Dance for Parkinson's: a new framework for research on its physical, mental, emotional, and social benefits. Complement Ther Med.

[27] Hashimoto H, Takabatake S, Miyaguchi H, Nakanishi H, Naitou Y (2015). Effects of dance on motor functions, cognitive functions, and mental symptoms of Parkinson's disease: a quasi-randomized pilot trial. Complement Ther Med.

[28] Faul F, Erdfelder E, Buchner A, Lang A (2009). Statistical power analyses using G*Power 3.1: tests for correlation and regression analyses. Behav Res Methods.

[29] Cohen J (1988). Statistical power analysis for the behavioral sciences (2nd ed.).

[30] Lötzke D, Ostermann T, Büssing A (2015). Argentine tango in Parkinson disease--a systematic review and meta-analysis. BMC Neurol.

[31] McKay JL, Ting LH, Hackney ME (2016). Balance, Body Motion, and Muscle Activity After High-Volume Short-Term Dance-Based Rehabilitation in Persons With Parkinson Disease: A Pilot Study. J Neurol Phys Ther.

[32] Earhart GM, Duncan RP, Huang JL, Perlmutter JS, Pickett KA (2015). Comparing interventions and exploring neural mechanisms of exercise in Parkinson disease: a study protocol for a randomized controlled trial. BMC Neurol.

[33] Kiepe M, Stöckigt B, Keil T (2012). Effects of dance therapy and ballroom dances on physical and mental illnesses: A systematic review. The Arts in Psychotherapy.

[34] Pisu M, Demark-Wahnefried W, Kenzik KM, Oster RA, Lin CP, Manne S, Alvarez R, Martin MY (2017). A dance intervention for cancer survivors and their partners (RHYTHM). J Cancer Surviv.

[35] Barrett FS, Janata P (2016). Neural responses to nostalgia-evoking music modeled by elements of dynamic musical structure and individual differences in affective traits. Neuropsychologia.

[36] Hwang PW, Braun KL (2015). The Effectiveness of Dance Interventions to Improve Older Adults' Health: A Systematic Literature Review. Altern Ther Health Med.

[37] McKee KE, Hackney ME (2013). The effects of adapted tango on spatial cognition and disease severity in Parkinson's disease. J Mot Behav.

[38] Brauer SG, Woollacott MH, Lamont R, Clewett S, O'Sullivan J, Silburn P, Mellick GD, Morris ME (2011). Single and dual task gait training in people with Parkinson's disease: a protocol for a randomised controlled trial. BMC Neurol.

[39] Capato TT, Tornai J, Ávila P, Barbosa ER, Piemonte ME (2015). Randomized controlled trial protocol: balance training with rhythmical cues to improve and maintain balance control in Parkinson's disease. BMC Neurol.

[40] Nilsson MH, Iwarsson S (2013). Home and health in people ageing with Parkinson's disease: study protocol for a prospective longitudinal cohort survey study. BMC Neurol.

[41] Dorsey ER, Achey MA, Beck CA, Beran DB, Biglan KM, Boyd CM, Schmidt PN, Simone R, Willis AW, Galifianakis NB, Katz M, Tanner CM, Dodenhoff K, Ziman N, Aldred J, Carter J, Jimenez-Shahed J, Hunter C, Spindler M, Mari Z, Morgan JC, McLane D, Hickey P, Gauger L, Richard IH, Bull MT, Mejia NI, Bwala G, Nance M, Shih L, Anderson L, Singer C, Zadikoff C, Okon N, Feigin A, Ayan J, Vaughan C, Pahwa R, Cooper J, Webb S, Dhall R, Hassan A, Weis D, DeMello S, Riggare SS, Wicks P, Smith J, Keenan HT, Korn R, Schwarz H, Sharma S, Stevenson EA, Zhu W (2016). National Randomized Controlled Trial of Virtual House Calls for People with Parkinson's Disease: Interest and Barriers. Telemed J E Health.

[42] Ashburn A, Roberts L, Pickering R, Roberts H, Wiles R, Kunkel D, Hulbert S, Robison J, Fitton C (2014). A Design to Investigate the Feasibility and Effects of Partnered Ballroom Dancing on People With Parkinson Disease: Randomized Controlled Trial Protocol. JMIR Res Protoc.

