# Supplementary figures and images for: Brazilian Samba Protocol for Individuals With Parkinson’s Disease: A Clinical Non-Randomized Study
Source: JMIR Res Protoc. 2017 Jul 4;6(7):e129. doi: 10.2196/resprot.6489 (PMC5516099; doi:10.2196/resprot.6489)

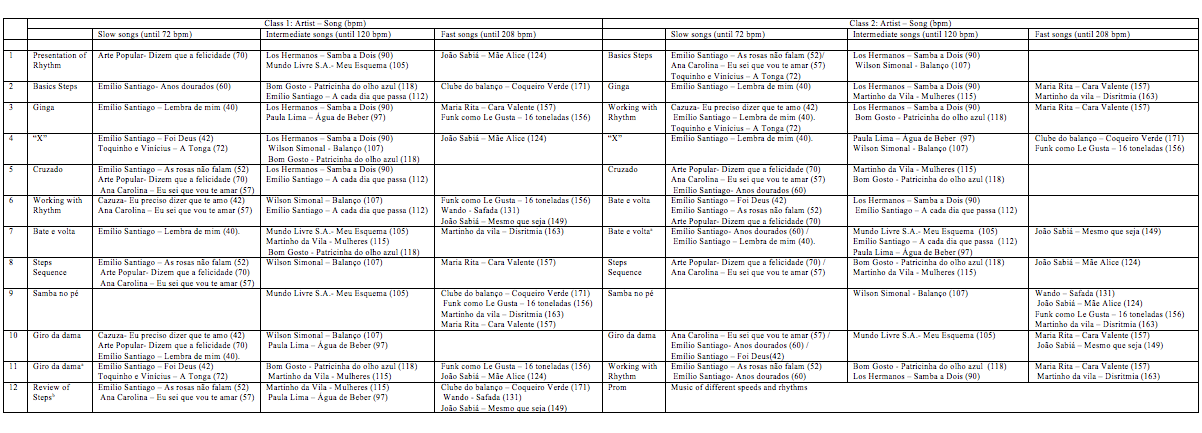

Supplement: Multimedia Appendix 1 [file resprot_v6i7e129_app1.png]
